# Microwave synthesis and actuation of shape memory polycaprolactone foams with high speed

**DOI:** 10.1038/srep11152

**Published:** 2015-06-08

**Authors:** Fenghua Zhang, Tianyang Zhou, Yanju Liu, Jinsong Leng

**Affiliations:** 1 Centre for Composite Materials and Structures, Harbin Institute of Technology (HIT), Harbin 150080, P. R. China; 2 Department of Astronautical Science and Mechanics, Harbin Institute of Technology (HIT), Harbin 150001, P. R. China

## Abstract

Microwave technology is a highly effective approach to fast and uniform heating. This article investigates that the microwave heating as a novel method is used to rapidly foam and actuate biocompatible and biodegradable shape memory crosslinked-polycaprolactone (c-PCL) foams. The optical microscope proves that the resulting c-PCL foams have homogenous pore structure. Mechanical behavior and shape memory performance of c-PCL foams are investigated by static materials testing. Shape recovery ratio is approximately 100% and the whole recovery process takes only 98 s when trigged by microwave. Due to the unique principle of microwave heating, the recovery speed of c-PCL foams in microwave oven is several times faster than that in hot water and electric oven. Hence compared to the traditional heating methods, microwave is expected to bring more advantages to modern industry and scientific research in the field of smart materials and structures.

Shape-memory polymers (SMPs) have been defined as promising smart materials because of their extraordinary qualities since the 1980 s^1^,^2^. SMPs are capable of remembering their temporary shapes and returning to their original shapes in response to the stimuli^3^ (heat^4^, electricity^5^, light^6^, magnetism^7^, moisture^8^ and even a change in PH value^9^). Due to their special functions, SMPs are the ideal choice for an intelligent system which can be served as hinges, morphing skin, and smart clothes^10^,^11^, etc. It is the flexibility and phase change of the SMPs that makes the deformation of SMPs possible^12^,^13^,^14^. However, the lack of structural diversity of SMPs restricts the potential applications. The porous SMP foams exhibit unique properties, such as light weight, high expansion volume, material saving impact resistance, dielectric and thermal resistance^15^,^16^,^17^. Features of SMP foams provide access to new utility in various fields^18^,^19^,^20^, including mini invasive surgery in biomedical field^21^,^22^ and self-deployable structures in aerospace engineering^23^, and so on.

From the practical applications, biocompatible and biodegradable SMP foams have been explored for various biomedical applications^24^,^25^, especially in aneurysm treatment^26^. Polycaprolactone (PCL) foams that are a kind of aliphatic thermoplastic polyester are biocompatible, biodegradable and elastic^27^. Sosnik A· *et al.* reported that the molecular weight increasing enable the melting temperature of PCL to change from 43 °C to 60 °C^28^. It was proved by Sun H and co-works that the foam scaffold was easy to be compressed into a small volume and then recovered to its original shape when trigged by certain stimuli. It could be degraded after the tissue regenerates^29^. Coombes A·G· *et al.* found that shape memory PCL -PEG foams could be triggered at body temperature and had the potential applications in scaffolds and regenerative medicine^30^. PCL cross-linked efficiently by benzoyl peroxide (BPO) that shows shape memory performance has been reported^31^,^32^,^33^,^34^. For example, Han and co-workers investigated the effect of peroxide crosslinking on thermal and mechanical behaviour of PCL^31^. Furthermore, Shaobing Zhou *et al.* demonstrated the PCL cross-linked by BPO possessing excellent shape memory effect and the addition of functional particles in the crosslinking matrix made the PCL composite activated by electric or magnetic field^33^,^34^.

Recently, more and more foaming processes have emerged to fabricate PCL foams, including gas blowing, selective laser sintering, solid state supercritical fluids foaming, particulate leaching and phase separation^35^,^36^,^37^. According to Quadrini F· and co-workers, the homogenous foams were achieved by means of particulate leaching method which was to add urea particulates in polymer matrix^38^. Solid state foaming using supercritical CO_2_ was explored by Jenkins M·J· and Salerno A· *et al*^39^,^40^,^41^,^42^. Phase separation to obtain the foams is clean and simple. However, these methods have some disadvantages. The temperature gradient and hysteresis effects often lead to non-uniform distribution and local overheating resulting in the partial material mechanical failure. In addition, the process is time consuming and very complicated.

Microwave technology is considered to be a highly efficient, rapid and uniform heating source. Due to these merits, microwave has been utilized in comprehensive and advanced systems, such as food, rubber, sterilization, measurement, medical treatment, microwave plasma and chemical industry^43^. In addition, microwave heating without temperature gradient and hysteresis effects is available to heat materials in molecular level and to exhibit fast response, which is an excellent way to meet the high requirements of uniform heating. To advance the actuation speed of SMPs, microwave is also needed. Microwave not only heats fast but also is capable of realizing the long distance control, which provides a new stimulus to actuate the active morphing polymers.

In this paper, we describe the synthesis and actuation of cross-linked PCL foams by microwave which is an easy and fast approach. The test results demonstrate that c-PCL foams exhibit excellent shape memory effect. The resulting foams are triggered by microwave oven, electronic oven and water bath respectively. And the shape recovery speed in microwave oven is the fastest. Because of the uniform and high speed heating, the microwave is a promising technology that can be employed to expand the stimuli methods in smart responsive materials.

## Results

### Morphology and porous structure

**Figure 1:**
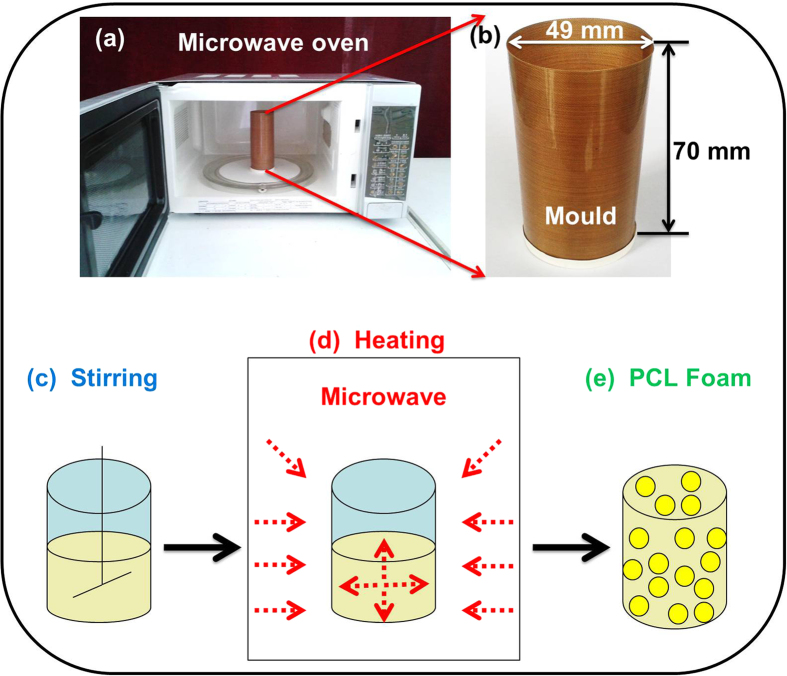
Process of microwave heating and synthesis of PCL.

The resulting c-PCL foams were fabricated by means of microwave. Figure 1 shows the equipment, materials and process to fabricate the PCL foam. As shown in Fig. 1a, the microwave oven for home use purchased from the Chinese company Galanz, was applied as heating source. It was modified simply to record the shape recovery process of c-PCL foams under microwave radiation. Figure 1b shows the mould is designed as a cylindrical object (49 mm diameter × 70 mm height) fabricated by release films. The mixture is stirred to prepare the uniform solution (Fig. 1c). Figure 1d shows the microwave heating process: microwaves passed through the media, dielectric loss caused the increase of temperature. The microwave heating is from inside to outside to make the samples heated uniform. This step played an enormous role in foaming. At last, we obtained the PCL foam (Fig. 1e) with a uniform pore distribution which was used for the following characterization.

**Figure 2:**
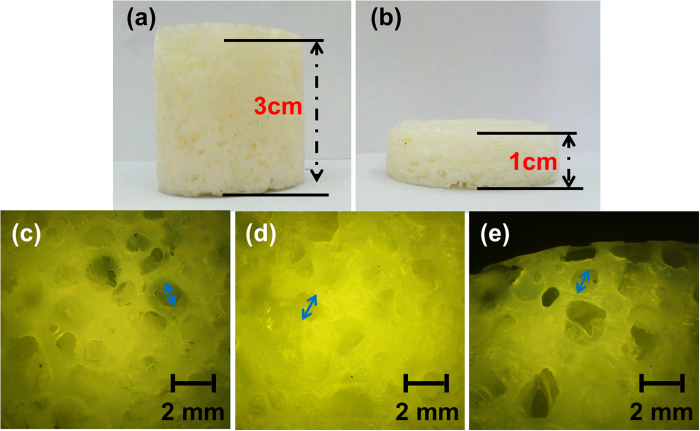
Samples of c-PCL foams: (**a**) is original shape, (**b**) is the shape after compression; (**c**), (**d**), and (**e**) are optical microscope images of c- PCL foams at × 20 magnification with 10%, 15%, 20% BPO, respectively.

The mechanism was that the dichloromethane as physical foaming agent was able to induce lots of bubbles in PCL system and then the microwave heating made the PCL crosslink and cure. The original c-PCL foam and the compressed foam are shown in Fig. 2a,b. It was observed that c-PCL foam had large compressive deformation. The morphology and pore distribution of these foams were characterized by optical microscope. BPO, as an initiator used to form the cross-linked structure, played an important role in the shape memory effect of c-PCL foams. Because PCL was a kind of linear polymer, it failed to show shape memory effect without cross-linked network. The “net points” of crosslinking determined the permanent shapes of c-PCL foams. Figure 2c–e show the optical images of c-PCL foams with the addition of 10%, 15% and 20% BPO respectively. At a 20 magnification, the optical microscope images illustrated that the foams had homogeneous porous structure.

The porosity of the foams was assessed by the following equation (1)^42^:


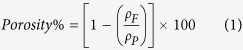


where *ρ*_p_ is the density of the polymeric phase (pure PCL). 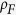 is the density of the PCL foam.

The number of pores per cm^3^ of the foam was assessed by the equation (2)^44^:


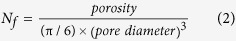


where *N*_*f*_ is the number of pores per cm^3^ of the foam.

Table 1 presents the performance parameters of c-PCL foams, including the density, porosity, and the number of pores per cm^3^ of the foam (N_f_).

### Thermal properties

**Figure 3:**
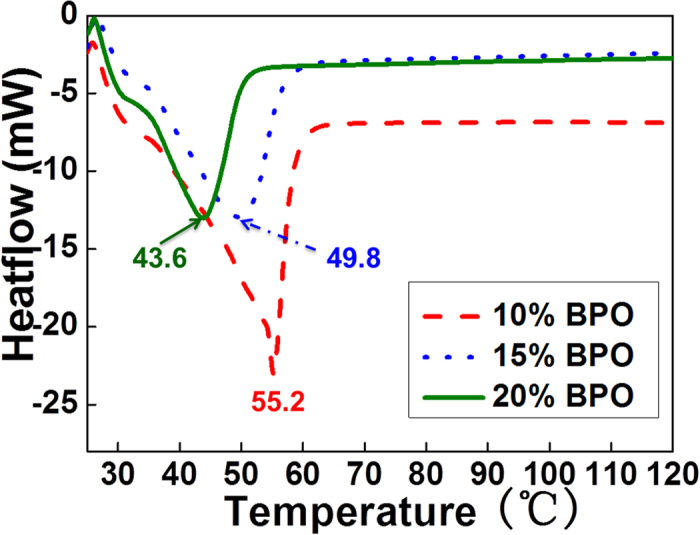
DSC curves of c-PCL foams with different contents of BPO.

The transition temperature that was analyzed by differential scanning calorimetry (DSC) played a critical role in shape deformation and shape recovery process of SMPs. As shown in Fig. 3, curves describe the melting peak of the c-PCL foams with 10% ~ 20% BPO. The melting temperature (*T*_*m*_) of c-PCL foams with 10% BPO was 55.2 °C. Furthermore, the *T*_*m*_ of c-PCL foams reduced to 49.8 °C and 43.6 °C with the addition of BPO increasing to 15% and 20% respectively. The cross-linked structure changes contributed to the melting temperature decrease. The underlying reason was that the crosslinking points in network structure destroyed the regularity of PCL molecular chains and limited the crystallization. However, the crystal structure could not be changed during this process. Table 2 shows the heat of fusion, degree of crystallinity and melting temperature of cross-linked PCL foams with different contents of BPO. The total enthalpy method was used to calculate the degree of crystallinity which was assessed by the equation (3)^31^:


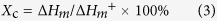


where *X*_c_ is the degree of crystallinity, Δ*H*_m_ is the specific enthalpy of melting, and Δ*H*_m_^+^ is the specific enthalpy of melting for 100% crystalline PCL, which is taken as 136 J·g^−1^ as reported in the literature^31^,^45^.

**Figure 4:**
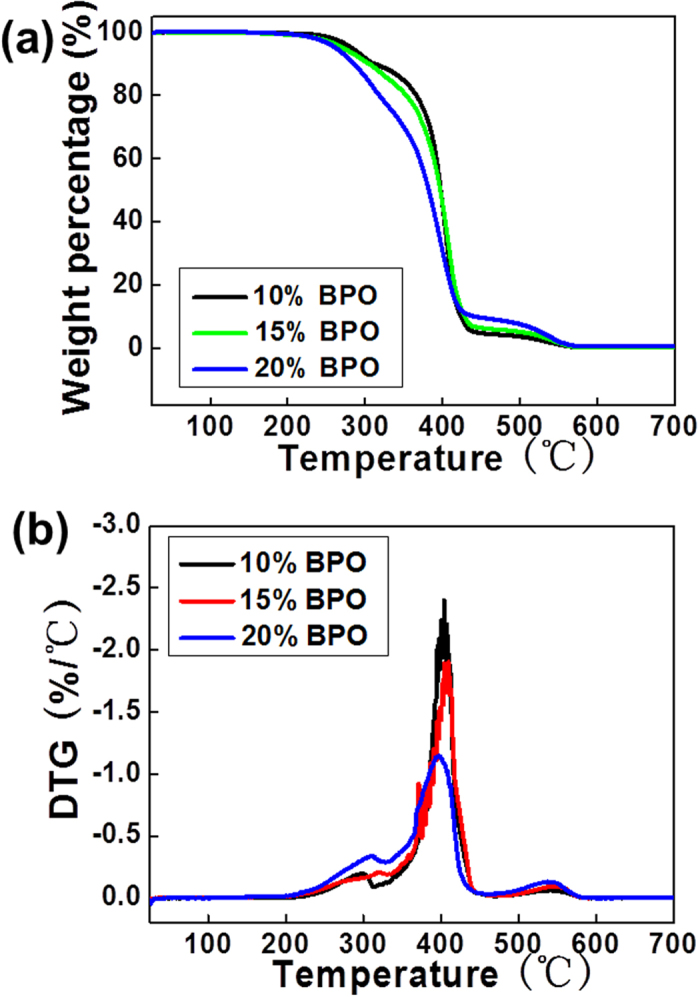
Thermal analysis images of c-PCL foams with different contents of BPO: (**a**) TGA curves and (**b**) DTG curves.

Thermal degradation of c-PCL foams was investigated by using the weight loss of specimens upon increasing temperature. TGA and DTG analysis are implemented for c-PCL foams as shown in Fig. 4a,b. TGA curves exhibited a weight loss from 210 °C to 310 °C and from 310 °C to 430 °C respectively. Accordingly, DTG curves displayed the main degradation peak at 395 °C and a shoulder at 290 °C, indicating that there were two consecutive mechanisms for this thermal degradation. The results demonstrated that the thermal degradation temperatures of three samples were slightly different. The degradation temperature reduced as a result of the crosslinking network.

### Crosslinking density

Cylindrical hydrogel constructs (5 mm in diameter and 5 mm in height) were fabricated from the PCL foams with different BPO contents for swelling. The constructs were swollen to equilibrium in chloroform at 20 °C for 72 h, weighed to determine the equilibrium swollen mass 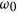

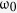
 and dried till constant mass of the constructs 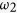
were obtained. The mass of solvent fraction after swelling is calculated by equation (4):


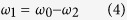


The equilibrium volumetric swelling ratio (*Q*) was calculated by equation (5)^46^:


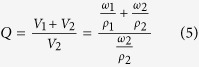


where is volume, 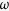 symbolize mass, *ρ* refers to density, subscript 1 stands for solvent while 2 for polymer.

The equilibrium volume fraction of polymer in the hydrogels 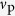 is:


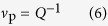


Flory-Rehner equation that based on the quilibrium swelling of polymer in solvent for three dimensional networks of randomly coiled chains was adopted^47^. The Flory-Rehner equation is given as:


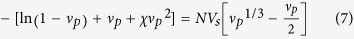


The average molecular weight between crosslinks is given as:


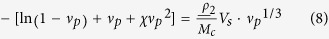


where N is the crosslinking density (mol/m^3^), *v*_s_ is the molar volume of the solvent chloroform, which is 80.7 ml/mol. Using −1.36 as the interaction parameter of *χ*.

The crosslinking density N and the average molecular weight of a chain between adjacent crosslinking points 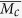 were calculated. As shown in Table 3, the crosslinking density increases with the increase of BPO.

### Thermal-mechanical properties and shape memory cycle**s**

**Figure 5:**
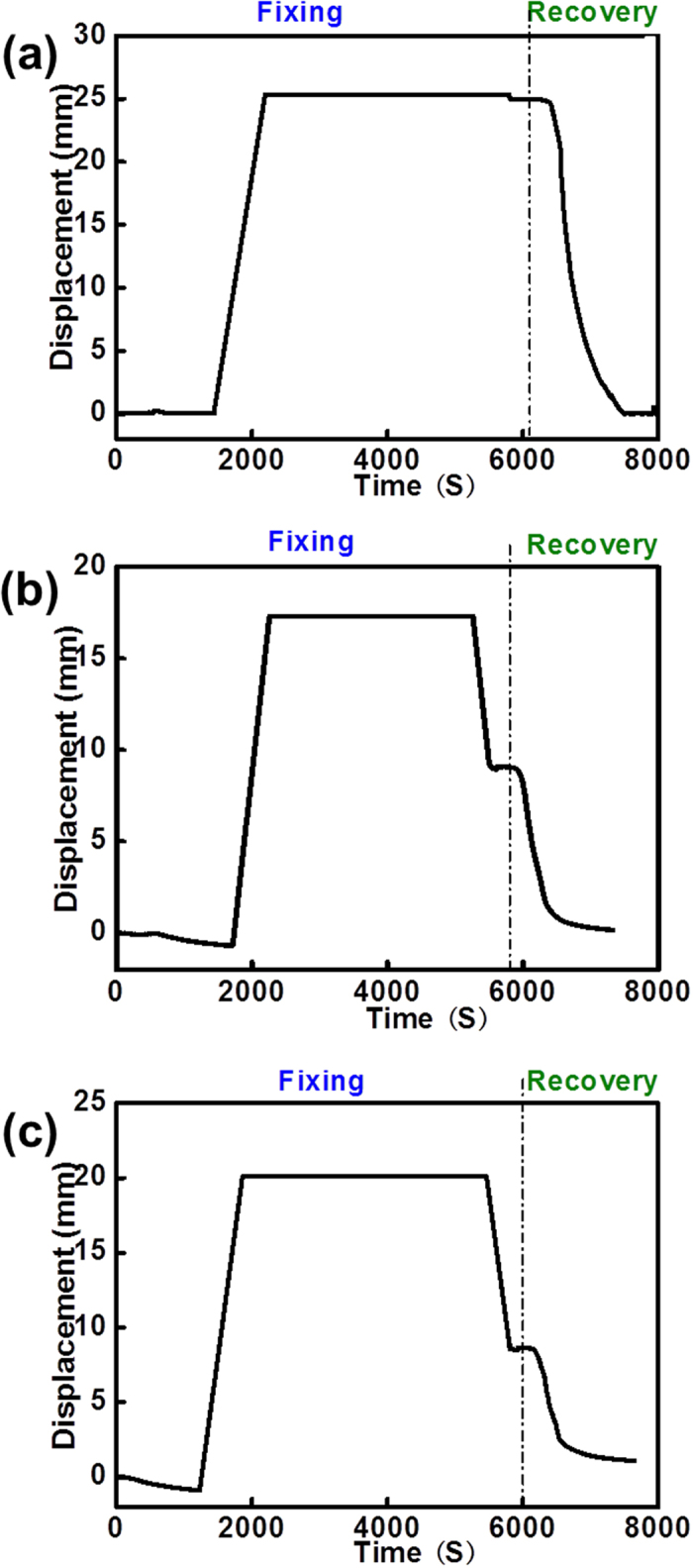
Shape memory performance of c-PCL foams with different BPO: (**a**) 10%, (b) 15% and (**c**) 20%.

Shape memory cycle obtained from static stretching machine was used to evaluate the shape memory performance (shape fixing ratio and shape recovery ratio) under compression model. The cylindrical c-PCL foams with 10%, 15% and 20% BPO were produced to characterize the shape memory effect. Each specimen was compressed to 50% strain at 100 °C (T > T_m_), and then cooled down to the room temperature and he temporary shape was fixed. Further, the recovery process was obtained when the sample was reheated to 100 °C. The time dependent displacements of compression and recovery are shown in Fig. 5. Figure 5a shows the displacement of shape memory process of c-PCL foam with 10% BPO which exhibits good shape fixing ratio after unloading. As shown in Fig. 5b,c, shape fixing ratios of the specimens with 15% BPO and 20% BPO have a little decrease when the external force is removed. It was clear from the Figure results that all of the curves were close to zero at the end of shape recovery cycle, which meant that c-PCL foams could fully return to their original shapes. According to the shape compressive deformation and shape recovery test, the shape fixity and shape recovery of c-PCL foams are shown in Table 4. It was observed that the shape fixing ratio sharply decreased from 98.7% to 43.0%. BPO content had an influence on shape memory effect because of the degree of PCL crystallinity changing. In this PCL system, the crystalline phase was served as switch unit to provide the shape fixity capacity. From the crystallinity results of PCL foams, the crystallinity decreased with the increase of BPO. Therefore, the shape fixity decreased with the increase of BPO. On the other hand, the cross-linked network was used as fixed phase to determine the shape recovery.

Equations (9) and (10) were used to calculate shape fixing ratio (*R*_f_) and shape recovery ratio (*R*_r_) for the shape memory behaviours^48^,^49^,^50^.


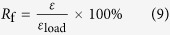


and


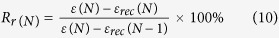


where *ε*_*load*_ was the maximum strain under load, *ε* was the fixed strain after cooling and load removal, and *ε*_*rec*_ was the strain after recovery.

**Figure 6:**
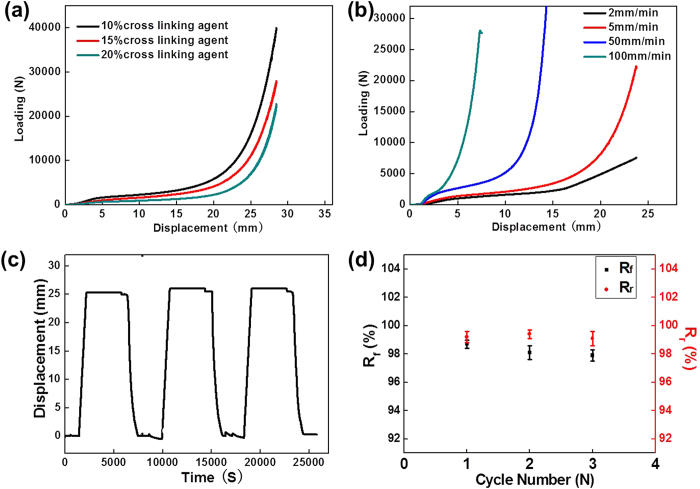
Compression test: (**a**) samples tested with different initiating agent; (**b**) samples tested under different compression speed; (**c**) three times shape memory cycle of c-PCL foam with 10% BPO and (**d**) shape recovery ratio and shape fixed ratio.

Figure 6a shows the static compression curves of c-PCL foams performed at room temperature and the difference mechanical properties. The dimension of PCL foam was 49 mm diameter × 30 mm height. The crosslinking reaction generated crosslinking points, which limited the movement of linear molecular chains. Due to the addition of BPO, the decreased crystallinity of PCL brought about the mechanical strength reduction, especially Young’s modulus. The elastic modulus and yield strength results are shown in Table 4. Under the thermal stimulus, the compressed foams could recover to their original shapes. As shown in Fig. 6b, the loading speed is faster and the strength of c-PCL foams is higher. Typically, the plastic deformation is accelerated at high speed, which leads to the higher energy storage and the higher strain hardening rate^49^,^50^.

Three consecutive cycles are carried out to evaluate the shape memory properties. As shown in Fig. 6c, the c-PCL foam with 10% BPO shows excellent shape memory performance. The melting temperature (*T*_m_) was 55.2 °C. It was compressed at *T*_m_ + 40 °C and then cooled down to room temperature. The temporary shape that was fixed during this process recovered to its original shape upon *T*_m_ + 40 °C. The R_f_ and the R_r_ above 98% in these cases (shown in Fig. 6d) confirm that continuous shape memory cycles almost do not affect the shape memory effect, and the foams are able to maintain the high shape fixing and shape recovery ratios. This feature effectively prolongs the service life for practical applications.

### Microwave actuated shape memory behavior

**Figure 7:**
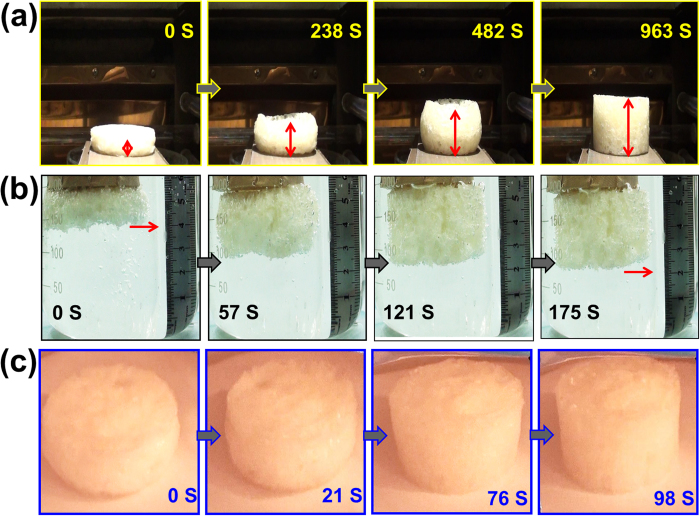
Shape recovery behavior of c-PCL foam in(**a**) water bath, (**b**) electrical oven and (**c**) microwave.

To evaluate the shape recovery performance, the specimen was compressed to the one-third height of original foam at 100 °C and held the shape for 24 hours at room temperature. The shape memory behaviour of c-PCL foam was stimulated upon different external conditions, including 100 °C water bath and 100 °C electrical oven. A typical heating-induced actuation is shown in Fig. 7a and the full recovery process takes 963 s in electronic oven. The temporary shape of c-PCL foam is able to return to its original shape in water bath after 175 s (Fig. 7b). Due to the different heat transfer rates, the foam in water bath recovered much faster than that in electronic oven.

A video camera is fixed to record the shape recovery of c-PCL foam triggered by microwave, which is shown in Fig. 7c. The deformed foam was subsequently heated and completely recovered. The entire process only took 98 s. The temporary shape recovered to the permanent shape was achieved, confirming that microwave radiation enabled the c-PCL foam to be actuated rapidly. And the actuation efficiency was improved by using microwave compared with other heating methods. Therefore, microwave is recognized as an ideal approach for quick response and actuation of shape memory c-PCL foams.

## Discussion

**Figure 8:**
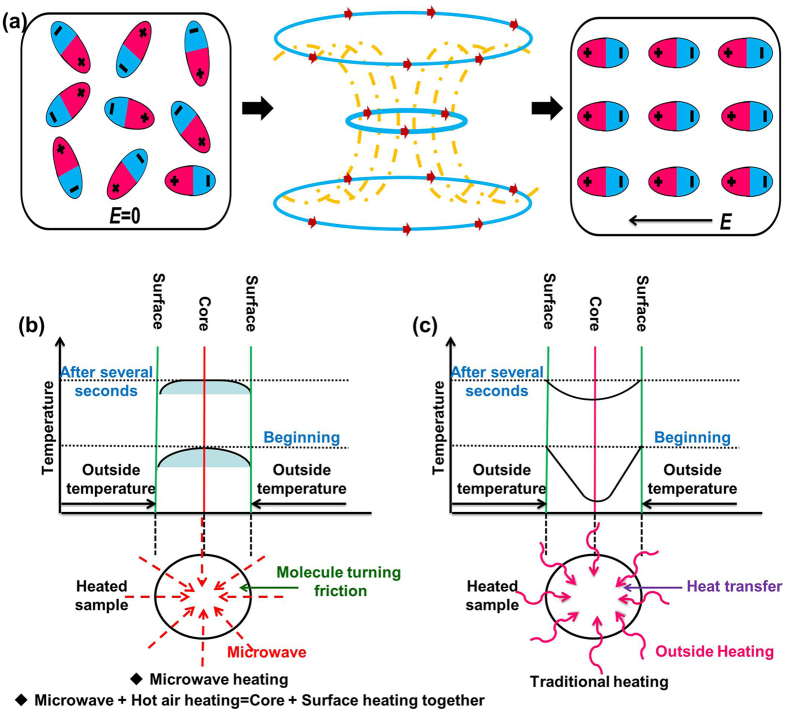
Mechanism of microwave heating (**a**), (**b**), and traditional heating (**c**).

The interactions between the polar molecules and the microwave electromagnetic field enable the microwave energy to be absorbed by samples^51^,^52^,^53^. As shown in Fig. 8a, the alternating magnetic field makes the random polar molecules orientation arrangement. During this process, the friction loss induced by polar molecules steering frequently converted the electromagnetic energy into heat. Figure 8b describes the microwave heating principle which is different from other heating methods. The microwave heating can radiate and penetrate the sample, which generates relatively uniform temperature distribution in the whole sample. However, the traditional heating from the surface to core is called surface heat-transfer method (shown in Fig. 8c) which makes the surface temperature higher. Microwave as the special heat source provides a route to greatly shorten the heat exchange time. The potential utility of microwave in stimulus responsive materials is expected in the future.

Microwave technology used to synthesize and actuate c-PCL foam was successfully investigated in this article. The obtained c-PCL foams exhibited homogenous pore structure and excellent shape memory behavior. The transition temperature decreased from 55.2 °C to 43.6 °C with the BPO increase. As the static compression speed increased, the strength of the c-PCL foam increased. It took the c-PCL foams only 98 s to fully recover to their original shapes in microwave oven, 2 times and 10 times faster than that in water bath and electric oven respectively. Shape memory c-PCL foams fabricated by microwave offer an opportunity for potential applications in smart materials and structures, especially in the biomedical treatments (tissue engineering, regenerative medicine, etc.) and the microwave-activated method also provides a high speed remote control.

## Methods

### Preparation of PCL foam via microwave

Poly(caprolactone) (PCL, CAPA 6500C, mean molecular weight 50 000) was purchased from Perstorp UK limited in pellet. Silicone DC-200 was supplied by Kermel. Dichloromethane (supplied by Tianjin Fuyu Fine Chemical Co., Ltd) was the solvent and physical foaming agent. Benzoyl peroxide (BPO supplied by Damao Chemical Reagent Factory) was used as initiating agent. The materials were used as received. Microwave output power and microwave field were 700 W and 2450 MHz respectively. The foams were produced by physical foaming including two steps. (1) We dissolved the PCL polymer particles into the dichloromethane along with a certain amount of simethicone and different amounts of BPO were added in PCL solution to enable the resulting foams with different degrees of crosslinking. Then the mixture was mechanically stirred (400 rpm for 3 hours) at room temperature. (2) When the mixture solution was homogeneous, it was poured into cylindrical moulds. The moulds as well as the mixture were put into the microwave oven for 4 minutes to produce the foams.

### Materials Characterization

Differential scanning calorimetric (DSC, Mettler) analysis was applied to test the melting temperature (*T*_m_) of the shape memory foams with different amount of BPO. The specimens were conducted from 25 °C to 120 °C at a heating rate of 10 °C/min in the air. The morphology and cellular structure of the c-PCL foams were investigated using an optical microscope (Keyence VHX-900) with the magnifying power of 20 ×. Thermo gravimetric analysis (TGA, Mettler) was carried out from 25 °C to 700 °C at a heating rate of 10 °C/min and an air flow rate of 40 mL/min.

### Shape memory effect

Static compression properties of c-PCL foams were quantified via a Zwick Roell testing system, working at the speed of 2, 5, 50, 100 mm/min respectively. Shape memory properties were performed in the same platform with a compression speed of 2 mm/min. The c-PCL foams were subjected to the following sequence: (1) compressed to 50% strain at *T*_m_ + 40 °C, (2) held at *T* < *T*_m_, (3) reheated to *T* > *T*_m_ to allow the free strain recovery. Three consecutive shape memory cycles were recorded. A small preload was applied to each sample to ensure that the sample touched the compression plates.

## Additional Information

**How to cite this article**: Zhang, F. *et al.* Microwave synthesis and actuation of shape memory polycaprolactone foams with high speed. *Sci. Rep.* **5**, 11152; doi: 10.1038/srep11152 (2015).

## Figures and Tables

**Table 1:** Performance parameters of c-PCL foams.

**Samples**	**Density/g·cm^−3^**	**Porosity**	***N*_*f*_/cm^−3^**
PCL foam with 10wt% BPO	0.40	63.55%	359.62
PCL foam with 15wt% BPO	0.37	66.39%	375.69
PCL foam with 20wt% BPO	0.40	63.89%	361.54

**Table 2:** Heat of fusion (Δ*H*_m_), degree of crystallinity (*X *_c_) and melting temperature (*T*_>m_) of cross-linked PCL foams.

**Samples**	**Δ*H*_m_/J·g^−1^**	***X*_c_/%**	***T*_m_/°C**
PCL foam with 10wt% BPO	42.4	31.2	55.2
PCL foam with 15wt% BPO	37.3	27. 4	49.8
PCL foam with 20wt% BPO	24.8	18.2	43.6

**Table 3:** Swelling ratio, Crosslinking density, and the average molecular weight between crosslinks of PCL foams with different BPO contents.

**Samples**	**Swelling ratio**	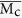 **(kg/mol)**	**Crosslinking density (mol/m^3^)**
PCL foam with 10wt% BPO	10.44	2.34	100.80
PCL foam with 15wt% BPO	9.21	1.89	135.87
PCL foam with 20wt% BPO	8.46	1.64	166.68

**Table 4:** Elastic modulus, yield strength (σ_f_), shape fixing and shape recovery ratio of PCL foams with different amounts of BPO.

**Samples**	**Elastic Modulus/MPa**	**σ_f_/MPa**	**Shape fixing ratio (*R*_f_)**	**Shape recovery ratio (*R*_r_)**
PCL foam with 10wt% BPO	7.18	0.86	98.7%	99.2%
PCL foam with 15wt% BPO	4.13	0.57	52.4%	98.1%
PCL foam with 20wt% BPO	2.54	0.33	43.0%	87.7%
